# [Retracted] ATP‑binding cassette transporter A7 accelerates epithelial‑to‑mesenchymal transition in ovarian cancer cells by upregulating the transforming growth factor‑β signaling pathway

**DOI:** 10.3892/ol.2024.14483

**Published:** 2024-06-03

**Authors:** Xia Liu, Qing Li, Jing Zhou, Su Zhang

Oncol Lett 16: 5868–5874, 2018; DOI: 10.3892/ol.2018.9366

Following the publication of this paper, it was drawn to the Editor's attention by a concerned reader that certain of the wound-healing assay data shown in Fig. 4C on p. 5872 were strikingly similar to data that had already been published in different form in another article written by different authors at a different research institute [Wang W and Wang J: Toll-like receptor 4 (TLR4)/cyclooxygenase-2 (COX-2) regulates prostate cancer cell proliferation, migration, and invasion by NF-κB activation. Med Sci Monit 24: 5588-5597, 2018].

Owing to the fact that the contentious data in the above article had already been published prior to its submission to *Oncology Letters*, the Editor has decided that this paper should be retracted from the Journal. The authors were asked for an explanation to account for these concerns, but the Editorial Office did not receive a reply. The Editor apologizes to the readership for any inconvenience caused.

